# Arginine Methylation by PRMT1 Regulates Muscle Stem Cell Fate

**DOI:** 10.1128/MCB.00457-16

**Published:** 2017-01-19

**Authors:** Roméo Sébastien Blanc, Gillian Vogel, Xing Li, Zhenbao Yu, Shawn Li, Stéphane Richard

**Affiliations:** a Terry Fox Molecular Oncology Group and Segal Cancer Center, Bloomfield Center for Research on Aging, Lady Davis Institute for Medical Research, and Departments of Oncology and Medicine, McGill University, Montréal, Québec, Canada; b Department of Biochemistry, Schulich School of Medicine and Dentistry, Western University, London, Ontario, Canada

**Keywords:** PRMT1, muscle regeneration, Eya1/Six1, MyoD, cell fate, muscle stem cell, Eya1

## Abstract

Quiescent muscle stem cells (MSCs) become activated in response to skeletal muscle injury to initiate regeneration. Activated MSCs proliferate and differentiate to repair damaged fibers or self-renew to maintain the pool and ensure future regeneration. The balance between self-renewal, proliferation, and differentiation is a tightly regulated process controlled by a genetic cascade involving determinant transcription factors such as Pax7, Myf5, MyoD, and MyoG. Recently, there have been several reports about the role of arginine methylation as a requirement for epigenetically mediated control of muscle regeneration. Here we report that the protein arginine methyltransferase 1 (PRMT1) is expressed in MSCs and that conditional ablation of *PRMT1* in MSCs using Pax7^CreERT2^ causes impairment of muscle regeneration. Importantly, PRMT1-deficient MSCs have enhanced cell proliferation after injury but are unable to terminate the myogenic differentiation program, leading to regeneration failure. We identify the coactivator of Six1, Eya1, as a substrate of PRMT1. We show that PRMT1 methylates Eya1 *in vitro* and that loss of PRMT1 function *in vivo* prevents Eya1 methylation. Moreover, we observe that PRMT1-deficient MSCs have reduced expression of Eya1/Six1 target *MyoD* due to disruption of Eya1 recruitment at the *MyoD* promoter and subsequent Eya1-mediated coactivation. These findings suggest that arginine methylation by PRMT1 regulates muscle stem cell fate through the Eya1/Six1/MyoD axis.

## INTRODUCTION

Tissue regeneration is a dynamic process requiring the presence of resident adult stem cells capable of long-term quiescence and rapid activation in response to injury. Skeletal muscle stem cells (MSCs), also called satellite cells, promote regeneration following damage of their supporting cells, the myofibers. Extrinsic stimuli activate the myogenic program leading to MSC proliferation, commitment, and differentiation to repair the damaged fiber or for self-renewal to maintain the pool and preserve the regenerative capacity. MSC loss of function leads to complete abolition of the regeneration and subsequently to loss of the skeletal muscle integrity (1).

Arginine methylation is an evolutionarily conserved posttranslational modification catalyzed by the protein arginine methyltransferase (PRMT) family, composed of 9 members and subdivided in three types: type I enzymes, which catalyze asymmetrical dimethylation of the arginine guanidino group, and type II and type III enzymes, which catalyze symmetrical dimethylation and monomethylation, respectively (2–4). Although PRMTs have a preference for certain short motifs such as the RGG/RG motifs (5), many PRMTs have a different substrate specificity and deviate from this motif selection. In the past decade, the emergence of interest for arginine methylation roles has led to *in vivo* studies using mouse models. Depletion of the major type I (PRMT1) in mice leads to embryonic lethality (6, 7), and more recently the conditional removal of PRMT1 using Nestin-Cre has been shown to implicate asymmetrical arginine methylation in the process of myelination (8). PRMT2-null mice were shown to be hypophagic and lean, as STAT3 methylation is lost (9). PRMT3^−/−^ mice are smaller than wild-type mice, and this hypomorphic allele has mRNA translation defects (10). CARM1/PRMT4-deficient mice have defects in adipogenesis, T cell differentiation, and hematopoiesis (11–13). PRMT5 is essential for mouse development, and whole-body genetic deletion leads to embryonic lethality (14), while conditional knockout of PRMT5 using Nestin-Cre demonstrated a key role in regulating the p53 pathway during neurogenesis (15) and a role for PRMT5 in adult hematopoiesis maintenance was shown using Mx1-Cre (16). Mice with whole-body knockout of PRMT6 are viable; however, the mouse embryo fibroblasts undergo premature senescence (17). Type III enzyme PRMT7 (2) is required in mice for B cell differentiation by controlling germinal center formation (18) and for muscle stem cell regeneration (19). PRMT8-deficient mice have motor coordination defects, and PRMT8 was characterized as an arginine methyltransferase and a phospholipase in Purkinje cells (20). In humans, *PRMT7* mutations were recently identified in a genetic screen and linked to pseudohypoparathyroidism (21).

In the past few years, arginine methylation has been implicated in skeletal muscle regeneration *in vivo*. CARM1/PRMT4 methylates Pax7 to regulate asymmetric division of MSCs by recruiting the MLL1/2 complex at the kb −57 enhancer of *Myf5* gene (22). Moreover, PRMT5 and PRMT7 were both shown to regulate *Cdkn1a* expression in a p53-independent fashion, as arginine methylation is critical for MSC expansion and guarding against premature senescence, respectively (19, 23). We showed that PRMT7 regulates the levels of *Dnmt3b* mRNA and controls DMNT3b-mediated DNA methylation of the *Cdkn1a* promoter region (19).

PRMT1 is responsible for generating the majority of asymmetric dimethylation of arginine in mammals (4). Here, we performed a candidate peptide array with selective arginine-containing peptides of key factors involved in the myogenic differentiation and performed an *in vitro* methylation assay. We identified Eya1 peptides to be methylated *in vitro* by PRMT1. Eya1 is a phosphotyrosine phosphatase responsible for histone H2AX dephosphorylation during DNA repair (24). Importantly, it was shown to interact with the transcription factors of the Six family to regulate the genetic cascade responsible for muscle stem cell fate during myogenesis upstream of the determinant transcription factor Pax3, therefore regulating *Myf4*, *Myf5*, *Myf6*, *MyoG*, and *MyoD* (25, 26). In adults, ectopic expression of the Eya1/Six1 complex is able to reprogram muscle fibers from the slow-twitch type to the fast-twitch type (27). Importantly, Six1 was reported as a regulator of adult MSC regenerative capacity and self-renewal by modulation of MyoD and myogenin (28). The involvement of Eya1 in adult MSC physiology remains unknown.

Here, we report that mice with a conditional deletion of PRMT1 using *Pax7*^*creERT2*^ exhibit decreased muscle regeneration upon cardiotoxin injury. Their MSCs displayed defects in the myogenic differentiation program. Surprisingly, we observed that the PRMT1-depleted MSCs had increased proliferation, suggesting augmentation of their self-renewal capabilities. We identify Eya1 as an *in vivo* PRMT1 substrate. PRMT1 depletion prevented coactivation of Six1 target genes by Eya1, compromising the activation of *MyoD*. Our findings define a role for PRMT1 in MSCs and a new role for arginine methylation in MSCs.

## RESULTS

### PRMT1 is expressed in muscle stem cells and determines cell fate.

**FIG 1:**
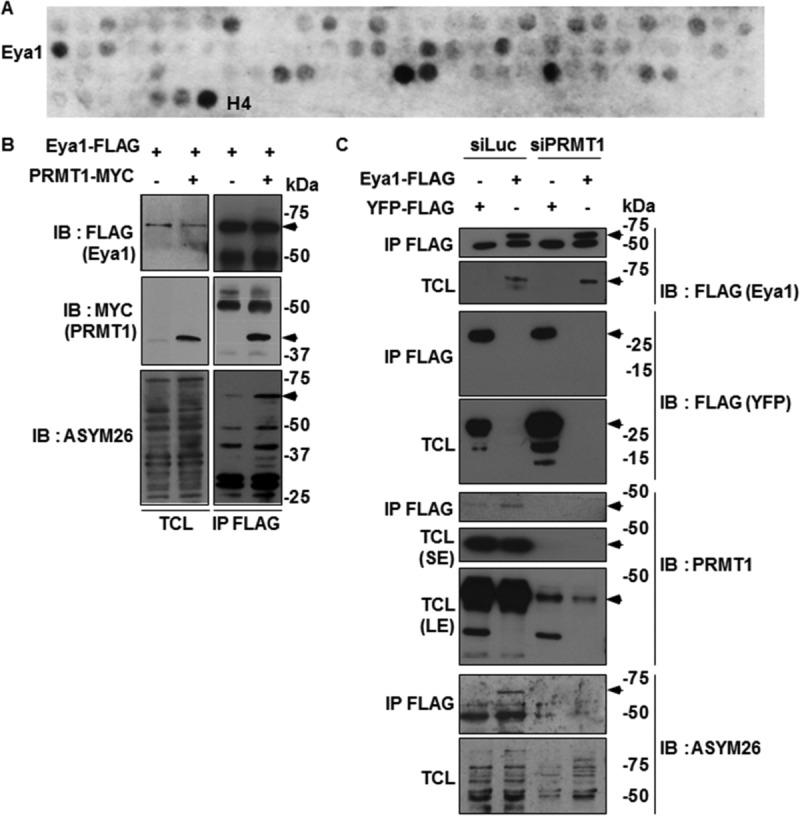
Eya1 is a PRMT1 substrate. (A) Candidate peptides were arrayed for major myogenic determinant proteins, and the filter was incubated with recombinant GST-PRMT1 and [^3^H]*S*-adenosylmethionine for *in vitro* methylation assay. The ^3^H-positive spots represent the methylated peptides. Peptides from histones H2B and H4 were used as positive controls. A complete list of the peptides is found in Table 1. (B) HEK293T cells were cotransfected with MYC epitope-tagged PRMT1 and FLAG-tagged Eya1 or FLAG-YFP as control. Cell lysates were prepared, and immunoprecipitations were performed with M2–anti-FLAG beads. The bound proteins were separated by SDS-PAGE and immunoblotted with anti-FLAG, MYC, and ASYM26. TCL, total cell lysates; IB, immunoblot; IP, immunoprecipitation. (C) HEK293T cells were transfected with siLuciferase (siLuc) or siPRMT1 for 24 h. Then, cells were transfected with expression vectors for FLAG-Eya1 or FLAG-YFP. Cell lysates were prepared 48 h later, and coimmunoprecipitations were performed as described for panel B. See also Tables 1 and 2.

Arginine methylation is a key posttranslational event required for MSC regenerative function (19). We asked whether the major type I enzyme, PRMT1, also plays a role in skeletal muscle regeneration. To identify myogenic factors that are substrates of PRMT1, we designed a candidate peptide array with selected candidates containing methylation motifs in their sequence, including peptides from Pax3, Pax7, Myf5, MyoD, Myf6, Pitx2, Six1/4, and Eya1/2, as well as proteins related to adult muscle homeostasis/regeneration, skeletal muscle disorders, and potential substrates predicted to be methylated on PRMT1 preferred motifs (RGG/RG and RXR motifs) (Table 1). The peptides listed in Table 2 were indeed methylated (preferred methylated motifs are in bold), and one of the top hits was one Eya1 peptide. We focused our work on Eya1, as it is known to play a key role in myogenesis (25). The peptide containing arginines 303, 305, 307, and 308, which are within the evolutionarily conserved eye-absent catalytic domain of Eya1, was methylated by PRMT1 in an *in vitro* methylation reaction (Fig. 1A; black arrow; peptide 2B). Eya1 is a dually specific tyrosine phosphatase and a cofactor of the Six family of transcription factors involved in the upstream regulation of the myogenesis cascade, including Pax3, MyoD, Myf5, MyoD, and Myf6 (25). Indeed, we confirmed the arginine methylation of Eya1 by cotransfecting FLAG epitope-tagged Eya1 with MYC epitope-tagged PRMT1 in HEK293T cells. Anti-FLAG immunoprecipitations were immunoblotted with anti-FLAG to confirm Eya1 immunoprecipitation (Fig. 1B, top panels), anti-MYC antibodies showed that PRMT1 coimmunoprecipitated with Eya1 (Fig. 1B, middle panels), and ASYM26 showed that Eya1 was indeed asymmetrically dimethylated by PRMT1 in cells (Fig. 1B, lower panels). Moreover, HEK293T cells were transfected with siLuciferase (siLuc; control) or siPRMT1 and the arginine methylation of FLAG-yellow fluorescent protein (FLAG-YFP) (control) or FLAG-Eya1 monitored by anti-FLAG immunoprecipitations followed by immunoblotting with ASYM26 (Fig. 1C). FLAG-Eya1 was methylated in siLuc-transfected cells but not in siPRMT1-transfected cells (Fig. 1C, lowest panels).

**TABLE 1:** Peptide arrays

Protein	Peptide identifier	Peptide sequence
Pax7	1A	PGMFSWEIRDRLLKDGHCD
Pax7	2A	PDLPLKRKQRRSRTTFTAEQ
Pax7	3A	QVWFSNRRARWRKQAGANQL
Pax3	4A	DLPLKRKQRRSRTTFTAEQL
Pax3	5A	QVWFSNRRARWRKQAGANQL
Myf5	6A	DRRKAATMRERRRLKKVNQA
MyoD	7A	DRRKAATMRERRRLSKVNEA
Myf6	8A	RRKAATLRERRRLKKINEA
MyoG	9A	KRKSVSVDRRRAATLREKRR
MyoG	10A	LNQEERDLRYRGGGGPQPGV
FOXO1A	11A	IDPDFEPLPRPRSCTWPLPR
FOXO1A	12A	PEGGKSGKSPRRRAASMDNN
FOXO1A	13A	SKFAKSRSRAAKKKASLQS
FOXO1A	14A	DNWSTFRPRTSSNASTISGR
FOXc2	15A	FENGSFLRRRRRFKKKDVS
Fgfr4	16A	RLAPAGRVRGWRGRLEIASF
Fgfr4	17A	HGENRIGGIRLRHQHWSLVM
Fgfr4	18A	LPLDPLWEFPRDRLVLGKPL
Fgfr4	19A	KGNLREFLRARRPPGPDLSP
Lbx1	20A	GKAAPGEERRRSPLDHLPPP
Pitx2	21A	GAEDPSKKKRQRRQRTHFTS
Pitx2	22A	ELEATFQRNRYPDMSTREE
Pitx2	23A	WTNLTEARVRVWFKNRRAKW
Pitx2	24A	FKNRRAKWRKRERNQQAELC
Six4	25A	KARYTEAERARGRPLGA
Six4	26A	VDKYRLRRKFPLPRTIW
Six4	27A	NWFKNRRQRDRNPSETQSK
Six1	28A	KLRGRPLGAVGKYRVRRKFP
Six1	29A	NWFKNRRQRDRAAEAKEREN
Eya1	30A	DSERLRRGSDGKSRGRGRRN
Eya1	1B	MRKLAFRYRRVKEIYNTYKN
Eya1	2B	RGSDGKSRGRGRRNNNPSP
Eya2	3B	RPHRASDGKLRGRSKRNSDP
Eya2	4B	MRKLAFRYRRVKEMYNTYRN
Meox2	5B	HHNYLTRLRRYEIAVNLDLT
Tbx1	6B	MDFVPVDDKRYRYAFHSSSW
Tbx1	7B	PGALPLVSAFARSRNPVASP
PKM1/2	8B	RSAHQVARYRPRAPIIAVTR
Dock7	9B	VRPASLNLNRSRSLSNSN
Dock7	10B	YDFTESHNQRGRPICIAPDD
Dock7	11B	IGARQEMVRRSRGQLERSPS
Dock7	12B	SAFGSQENLRWRKDMTHWRQ
CLCN1	13B	SENGGLQHRPRKDMGPRHNA
SmD3	14B	ILKAQVAARGRGRGMGRGNI
Fox1	15B	KRLHVSNIPFRFRDPDLRQM
Fox1	16B	FENSADADRAREKLHGTVVE
Fox1	17B	YRGAHLRGRGRTVYNTFRA
CUGBP	18B	VYEINILRDRSQNPPQSKG
MTMR1	19B	VNYYVRWNPRMRPQMPIHQN
FASN	20B	KKVIREPRPRSARWLSTSI
FASN	21B	LTQGEVYKELRLRGYDYGPQ
FASN	22B	CLRKEPGGHRIRCILLSNLS
FASN	23B	CMLGMEFSGRDRCGRRVMGL
FASN	24B	TTAYYSLVVRGRIQRGETVL
LaminA/C	25B	DRLAVYIDRVRSLETENAG
LaminA/C	26B	ETENAGLRLRITESEEVV
LaminA/C	27B	KTLDSVAKERARLQLELSKV
LaminA/C	28B	HEELQQSRIRIDSLSAQLSQ
LaminA/C	29B	KLRDLEDSLARERDTSRRLL
LaminA/C	30B	KEREMAEMRARMQQQLDEYQ
LaminA/C	1C	YRKLLEGEEERLRLSPSPT
LaminA/C	2C	PSPTSQRSRGRASSHSSQS
LaminA/C	3C	VDEEGKFVRLRNKSNEDQSM
LaminA/C	4C	GDPAEYNLRSRTVLCGTCGQ
CLTC1	5C	TDELVAEVEKRNRLKLLLPW
CLTC1	6C	LLILTAIKADRTRVMEYINR
Filamin-C	7C	VTEGCDPTRVRAFGPGLEGG
Filamin-C	8C	CSGPGLGTGVRARVPQTFTV
Filamin-C	9C	RMKESITRRRQAPSIATIGS
Filamin-C	10C	VFGDFLGRERLGSFGSITRQ
Cdk1	11C	VVYKGRHRVTGQIVAMKKI
PHB2	12C	PIIYDIRARPRKISSPTGSK
WDR77	13C	ERQLEAARYRSDGSLLLGVS
WDR77	14C	LSEVFRSRAHRDFVRDATWS
Bml	15C	RLLGSLWRHRPDSLDNTVQ
Bml	16C	ANQTRNERKRKKMSATHKPK
Bml	17C	KKMSATHKPKRRRTSYGGFR
IPO8	18C	MSNLGYLRARSCWVLHAFSS
FancD2	19C	MISKRRRLDSEDKENLT
FancD2	20C	DGVTSHVSRNRATEDGEDEA
Ash2l	21C	DKYWECMTTRQRPGKMTWPN
MEF2	22C	KKIQITRIMDERNRQVTFTK
MEF2	23C	RKINEDIDLMISRQRLCAVP
MEF2	24C	SIKSEPVSPPRDRTTTPSRY
MEF2	25C	DERESPSVKRMRLSEGWAT
CKM	26C	DPNYVLSSRVRTGRSIKGYT
CKM	27C	HPKFEEILTRLRLQKRGTGG
p38	28C	PPPGVSPSRLRIGDQEFDSL
p38	29C	TLIEPVARSRQGSGVILRQE
p38	30C	LPFKKGDILRIRDKPEEQWW
EZH2	1D	GPVCWRKRVKSEYMRLRQLK
EZH2	2D	KSEYMRLRQLKRFRRADEVK
EZH2	3D	KTPPKRPGGRRRGRLPNNSS
PCAF	4D	YWHLEAPSQRRLRSPNDDIS
PCAF	5D	NSHAPEEAKRSRVMGDIPVE
PCAF	6D	DLKTMSERLRNRYYVSKKLF
Histone H2B	7D	KKDGKKKKRSRKESY
Histone H4	8D	GRGKGGKGLGKGGAKRHRKV

**TABLE 2:** Peptide array candidates methylated by PRMT1

PRMT1 substrate	Peptide sequence^*a*^
Eya1	RGSDGKS**RGRGRR**NNNPSP
Fox1	FENSADAD**RAR**EKLHGTVVE
Bml	RLLGSLW**RHR**PDSLDNTVQ
Bml	ANQTRNE**RKR**KKMSATHKPK
Ash2l	DKYWECMTT**RQR**PGKMTWPN
Histone H4	**GRG**KGGKGLGKGGAK**RHR**KV

a Preferred methylated motifs are in bold.

**FIG 2:**
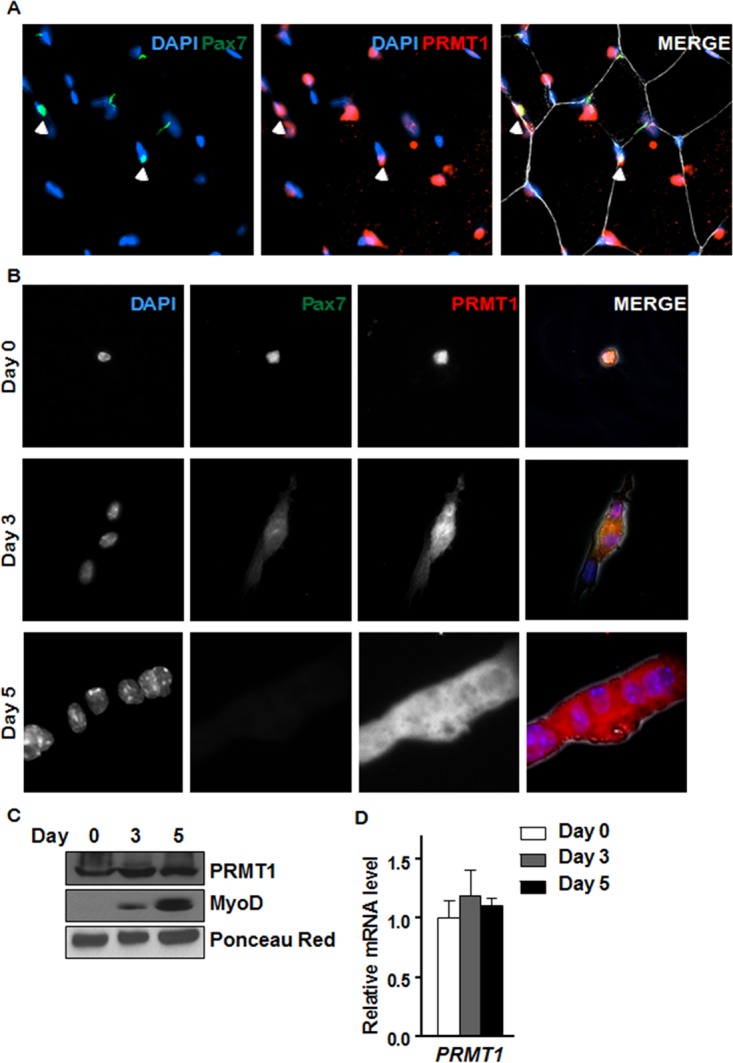
PRMT1 is expressed in quiescent and differentiating MSCs. (A) Immunofluorescence of tibialis anterior muscle cross-section from 6-week-old wild-type mice immunostained for PRMT1 and Pax7. The nuclei were stained with DAPI. (B) Differentiation assay of MACS-isolated MSCs fixed right after isolation (day 0) or cultured for 3 to 5 days prior to fixation, immunostained for PRMT1 and Pax7, and visualized by fluorescence microscopy. The nuclei were stained with DAPI. (C) Cell lysates were prepared from MACS-isolated MSCs 0, 3, and 5 days in culture and immunoblotted with anti-PRMT1 or anti-MyoD. Ponceau red staining of a band is shown to confirm equivalent loading. (D) RNA was extracted from MACS-isolated MSCs at 0, 3, and 5 days of culture, and the levels of *PRMT1* were measured by RT-qPCR and normalized to the average of *GAPDH*, *R18S*, and *TBP*.

We next examined the expression of PRMT1 in quiescent MSCs by costaining the tibialis anterior (TA) muscle from wild-type mice with anti-Pax7 antibodies, a marker of quiescent MSCs, and anti-PRMT1 antibodies followed by visualization by fluorescence microscopy. Pax7-positive MSCs costained with anti-PRMT1 antibodies (Fig. 2A). We next isolated MSCs and either used them immediately or allowed them to differentiate into mature myoblasts for 3 or 5 days. Immunostaining of the quiescent and mature myoblasts showed that PRMT1 was expressed at all stages of differentiation with both cytoplasmic and nuclear staining (Fig. 2B). We also observed that *PRMT1* mRNA and protein levels were at equal levels in quiescent cells (day 0) and in day 3 and 5 activated, proliferative cells and committed myoblasts (Fig. 2C and D). As controls, we showed that MyoD protein levels were increased with differentiation, while Ponceau red staining revealed equivalent loading (Fig. 2C).

**FIG 3:**
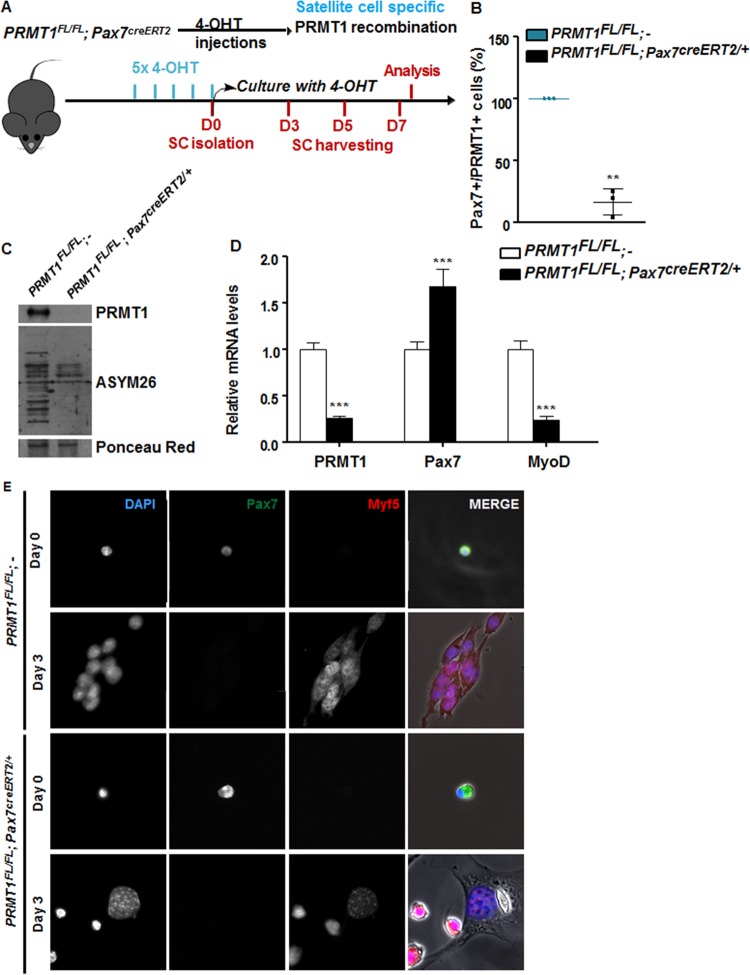
PRMT1-deficient muscle stem cells have myogenic defects. (A) Schematic representation of PRMT1 deletion in MSCs and work flow for MSC isolation and culturing. (B) MSCs were isolated and pooled from three *PRMT1*^*FL/FL*^; *Pax7Cre*^*ERT2/+*^ and three PRMT1^FL/FL^; − mice after 4-OHT injections. The cells were cultured for 3 days in medium supplemented with 50 nM 4-OHT prior to fixation and immunostaining for PRMT1 and Pax7 and visualized by fluorescence microscopy. (C) MSC lysates after 3-day culturing were separated by SDS-PAGE, and the levels of PRMT1-methylated proteins were determined by immunoblotting with anti-PRMT1 and ASYM26. Ponceau red staining of a band is shown to confirm equivalent loading. (D) Quantification of *PRMT1*, *Pax7*, and *MyoD* mRNA levels by RT-qPCR of the indicated MSCs 3 days after culturing. Values were normalized to the averages of *GAPDH*, *R18S*, and *TBP*. (E) Representative images of MSCs immunostained for Pax7 and Myf5 from days 0 and 3 after culturing. **, *P* < 0.01; ***, *P* < 0.001.

To define the role of PRMT1 in MSCs, we generated mice that are PRMT1 deficient using a 4-hydroxytamoxifen (OHT)-inducible CRE driven by the *Pax7* promoter. MSCs were isolated following five intraperitoneal injections in *PRMT1*^*FL/FL*^; *Pax7*^*creERT2/+*^ and wild-type (here designated *PRMT1*^*FL/FL*^; −) mice, the latter being used as controls (Fig. 3A). We confirmed the depletion of PRMT1 by immunostaining in Pax7-positive cells and observed a statistically significant decrease of Pax7^+^ PRMT1^+^ double-positive cells (*P* = 0.0015) (Fig. 3B) and reduced protein methylation in PRMT1-deficient MSCs (Fig. 3C). MSCs were cultured for 3 days in serum-rich medium to promote proliferation, and we observed that following PRMT1 depletion as visualized by reverse transcriptase PCR followed by quantitative PCR (RT-qPCR), the expression levels of *Pax7* increased while *MyoD* was significantly repressed, suggesting an impairment of the myogenic differentiation (Fig. 3D). Since *MyoD* mRNA levels were reduced, we assessed the commitment of MSCs with immunofluorescence using an anti-Myf5 antibody (Fig. 3E). Both quiescent MSCs isolated from wild-type (*PRMT1*^*FL/FL*^; −) and PRMT1-deficient (*PRMT1*^*FL/FL*^; *Pax7*^*creERT2/+*^) mice retained Pax7 expression, confirming that PRMT1 did not affect quiescent MSCs (Fig. 3E, day 0). After day 3 of culturing, wild-type (*PRMT1*^*FL/FL*^; −) activated MSCs entered the differentiation program, as they committed into proliferating progenitors, expressed Myf5, and aligned for fusion (Fig. 3E, second row). PRMT1-deficient MSCs at day 3 expressed Myf5 and not Pax7 but did not align for fusion (Fig. 3E; fourth row). These findings suggest that PRMT1-deficient MSCs have differentiation defects.

### PRMT1 controls Six1 transcriptional activity through Eya1 recruitment.

**FIG 4:**
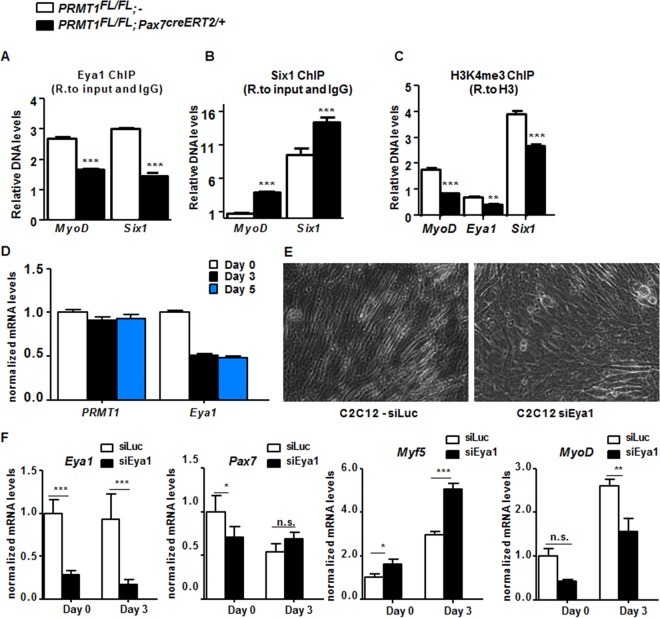
PRMT1 controls *MyoD* expression via mediation of Eya1/Six1 activity. (A) Relative (R.) levels of DNA from ChIP of Eya1 at *MyoD*, *Eya1*, and *Six1* upstream promoter region (approximately bp −500) normalized to input and IgG. (B) Relative levels of DNA from ChIP of Six1 at *MyoD*, *Eya1*, and *Six1* upstream promoter region (approximately bp −500) normalized to input and IgG. (C) Relative levels of DNA from ChIP of the histone mark H3K4me3 at *MyoD*, *Eya1*, and *Six1* upstream promoter region (approximately bp −500) normalized to H3. (D) C2C12 cell were differentiated for days 0, 3, and 5, and the mRNA levels of *PRMT1* and *Eya1* were measured by RT-qPCR and normalized to the average of *GAPDH*, *R18S*, and *TBP*. (E) A representative differential interference contrast image of C2C12 cultures after transfection with siLuciferase (siLuc) or siEya1 under differentiation conditions. (F) mRNA levels of *Eya1*, *Pax7*, *Myf5*, and *MyoD* were measured by RT-qPCR and normalized to *GAPDH*, *R18S*, and *TBP* in C2C12 transfected with siLuc or siEya1, harvested as undifferentiated (day 0) or after 3 days of culture in differentiation medium (day 3). *, *P* < 0.05; **, *P* < 0.01; ***, *P* < 0.001; n.s., not significant.

Eya1 is the coactivator of Six1 (27, 29), and the Six1/Eya1 complex can bind and regulate *MyoD* expression (30–32). We performed chromatin immunoprecipitation (ChIP) of Six1 and Eya1 at both *MyoD* and *Six1* promoter (Fig. 4A and B). The latter was used because it is known that Six1 associates with its own promoter (26, 32). Eya1 binding was reduced at *MyoD* and *Six1* genes in the absence of PRMT1 (Fig. 4A) (*P* < 0.001). In contrast, Six1 binding was increased at both *MyoD* and *Six1* genes in PRMT1-deficient cells (Fig. 4B) (*P* < 0.001). It was previously reported that Eya1 switches Six1-Dach activity from repressor to activator (29). Therefore, Six1 binding is likely to be repressive without Eya1, and indeed we observed decreased H3K4me3 at the *MyoD* and *Six1* promoters, consistent with gene repression (Fig. 4C) (*P* < 0.001).

The expression of PRMT1 and Eya1 mRNAs was examined in differentiating C2C12 cells. PRMT1 mRNA levels remained constant, while Eya1 mRNA levels decreased with differentiation (Fig. 4D). C2C12 treated with siEya1 had differentiation defects, while C2C12 cells transfected with siLuc did not (Fig. 4E and F). To further examine this differentiation defect, we assessed the expression of differentiation markers by RT-qPCR. *Eya1* expression was reduced in siEya1 cells, as expected, while *Myf5* was increased and *MyoD* decreased (Fig. 4F). These findings show that Eya1 is required for myogenic differentiation of C2C12, consistent with its role as a substrate of PRMT1.

### Absence of PRMT1 increases muscle stem cell expansion and long-term self-renewal but impairs differentiation.

**FIG 5:**
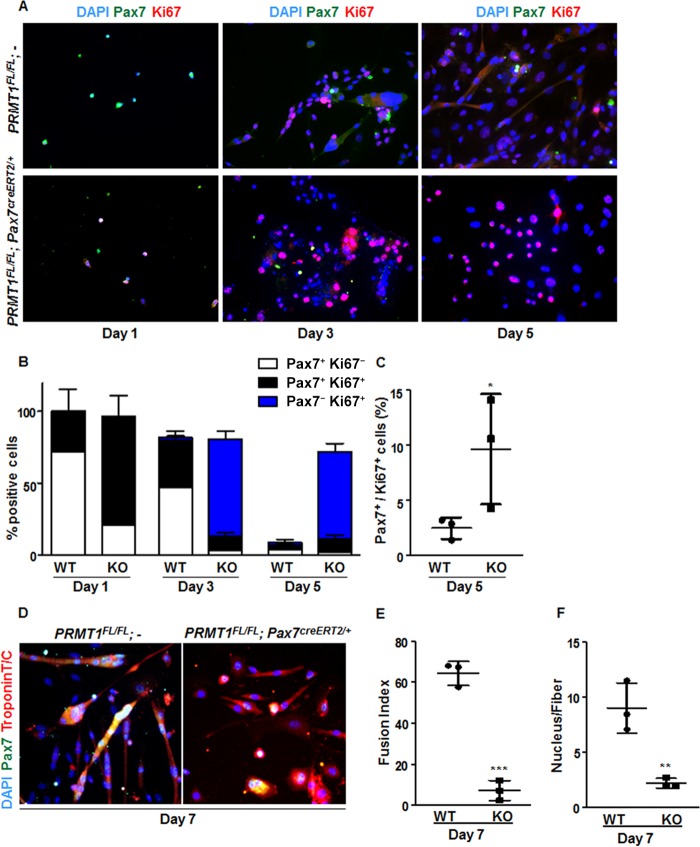
Cultured PRMT1-deficient MSCs display enhanced proliferation and impaired differentiation. (A) Representative images of MSCs immunostained for Pax7 (green) and Ki67 (red) and visualized by fluorescence microscopy. DAPI is blue. MSCs were cultured for 1, 3, or 5 days in proliferative medium. (B) Quantification of Pax7- and Ki67-positive cells as shown in panel A. For each condition, statistics were performed based on technical triplicates and counting >100 nuclei. Double-negative nuclei are not accounted for in this analysis. WT, wild type; KO, knockout. (C) Percentages of double-positive MSCs (Pax7^+^ Ki67^+^) after day 5 culturing with the indicated genotype. (D) Representative images of MSCs immunostained for Pax7 and troponin after 7 days of culture in differentiation medium. (E) The fusion index was calculated as the ratio of the number of myonuclei over the number of total nuclei in the field. For each condition, statistics were performed based on technical triplicates and counting >300 nuclei. (F) Average number of nuclei/fiber in each field after MSC day 7 of differentiation. For each condition, statistics were performed based on technical triplicates and counting >300 nuclei. This represents the ratio of the number of myonuclei over the total number of myofibers present in the field. *, *P* < 0.05; **, *P* < 0.01; ***, *P* < 0.001.

Given the role of Six1/Eya1 in myogenesis and adult MSC self-renewal (28) and the importance for MyoD during MSC differentiation (33), we wanted to evaluate the balance between proliferation and differentiation of PRMT1-deficient MSCs. The proliferative status of MSCs or their progeny was assessed by scoring cells positive for anti-Pax7 and anti-Ki67 or anti-Ki67 antibodies only, respectively. Only quiescent and self-renewing cells remain positive for anti-Pax7 immunostaining (1). We observed a significant increase in the number of PRMT1-deficient MSCs that stained for Ki67 compared to wild-type MSCs at days 1, 3, and 5 of culture (Fig. 5A and B). Actually, the majority of PRMT1-deficient MSCs repressed Pax7 expression at day 3 while being Ki67 positive (Fig. 5B, blue bar), suggesting that the absence of PRMT1 promotes proliferation. In contrast, at day 3, ∼50% of wild-type MSCs were mainly Pax7^+^ (Fig. 5B, white bars). After 5 days of culturing, most wild-type MSCs were differentiated, as they had fused as multinucleated myofibers and were both Pax7 and Ki67 negative (Fig. 5B). In contrast, ∼60% of PRMT1-deficient MSCs were Pax7^−^ Ki67^+^, suggesting that the absence of PRMT1 maintains MSCs in a persistent proliferative state (Fig. 5B, blue bar). Notably, after 5 days in culture, PRMT1-depleted MSCs significantly promoted the expansion of Pax7^+^ Ki67^+^ cells (*P* = 0.467), suggesting that even in the absence of PRMT1, progenitors are capable of self-renewal by maintaining a Pax7-positive pool of cells (Fig. 5C) (∼12%).

As the absence of PRMT1 maintained MSCs in proliferation (Fig. 5A to C) and decreased *MyoD* expression, we assumed that myogenic differentiation was compromised. Differentiation was assessed by expression of troponin T/C, and we scored the ability of the MSC progenitors to form mature multinucleated fibers. We found that PRMT1-deficient MSCs were impaired in generating fused myotubes compared with wild-type MSCs on differentiation day 7 (Fig. 5D). The fusion index was drastically reduced in PRMT1-null MSCs (*P* < 0.001), with <10% of cells having 2 or 3 nuclei per fiber (*P* = 0.007) (Fig. 5E and F). PRMT1 loss of function impairs MSC differentiation and maintain MSCs in the proliferation and self-renewal state. Together, these results emphasize a role for PRMT1 in the regulation of MSC cell fate decision between MSC proliferation and differentiation.

### Mice with PRMT1-deficient MSCs have muscle regeneration defects.

**FIG 6:**
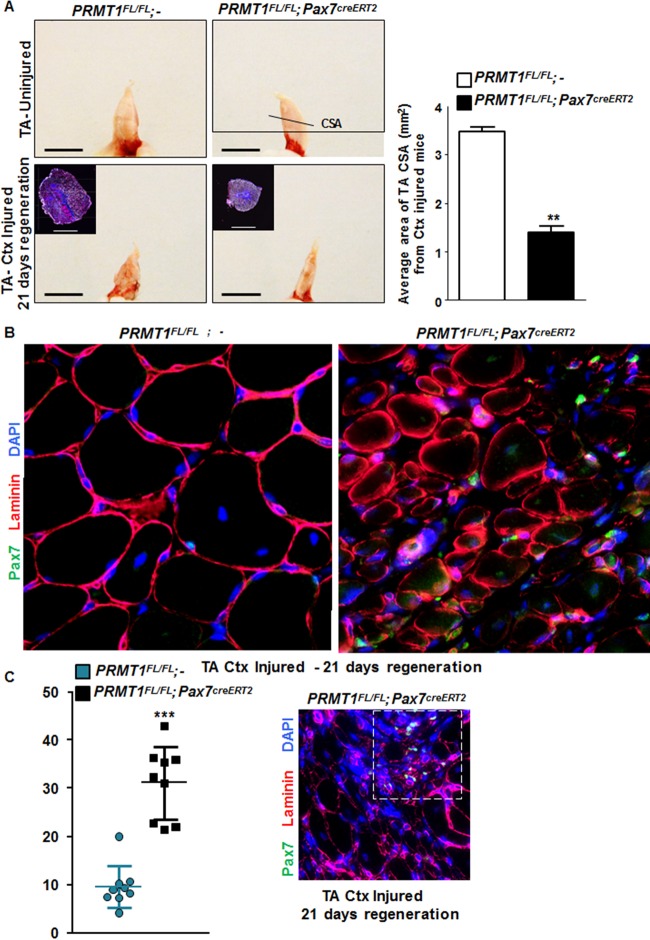

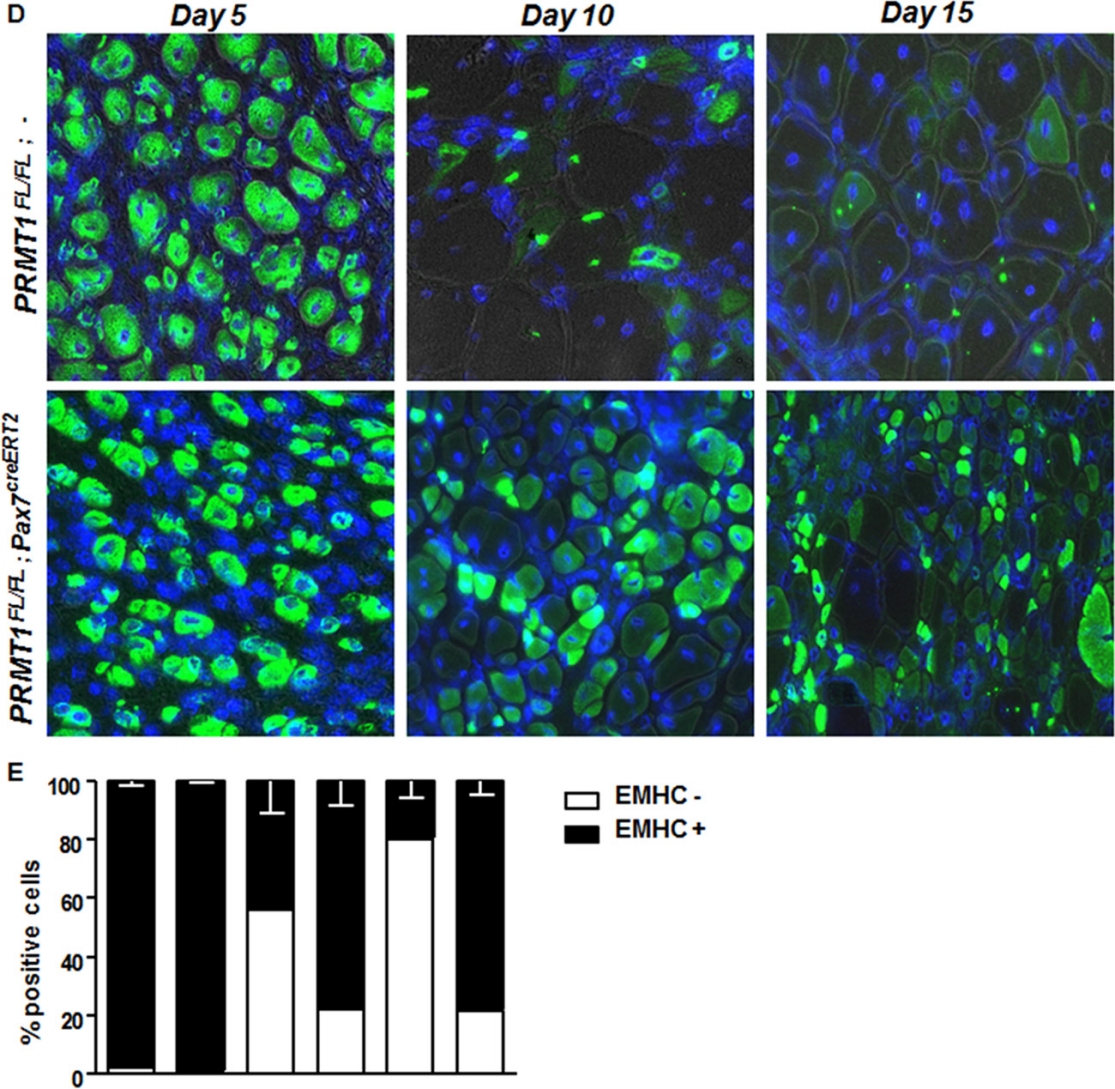
Adult *PRMT1*^*FL/FL*^; *Pax7Cre*^*ERT2/+*^ mice have impaired muscle regeneration following cardiotoxin-induced injury. (A) (Left) Representative images of injured or uninjured tibialis anterior (TA) from PRMT1^FL/FL^; Pax7Cre^ERT2/+^ and PRMT1^FL/FL^; − mice extracted 21 days after Ctx injection. The injured TA muscles were cryosectioned at the cross-section area (CSA) (lower images, top left corner), which was measured using nine mice of each genotype. White bar, 100 μm; black bar, 1 cm. (Right) For each mouse, five cross sections were analyzed and the average was calculated to be representative of the samples. (B) The injured TA muscles of the indicated genotypes were cryosectioned, freshly fixed, and immunostained for Pax7 and laminin to assess regeneration. (C) (Left) Quantification of the Pax7-positive cells per fiber after regeneration in PRMT1^FL/FL^; Pax7Cre^ERT2/+^ and PRMT1^FL/FL^; − mice TA cross sections; (right) representative image from Pax7Cre^ERT2/+^ and PRMT1^FL/FL^; − TA section after regeneration to show a hot spot of proliferative Pax7-positive cells. (D) Injured TA cross sections from PRMT1^FL/FL^; Pax7Cre^ERT2/+^ and PRMT1^FL/FL^; − mice extracted at days 5, 10, and 15 after Ctx injection. Injured TA were cryosectioned at the cross section area and stained for embryogenic myosin heavy chain (EMHC) to index regeneration. For each time point, three mice of each genotype were used. (E) Quantification of the EMHC-positive and -negative regenerating fibers in PRMT1^FL/FL^; Pax7Cre^ERT2/+^ and PRMT1^FL/FL^; − TA cross sections. For each time point, three mice of each genotype were used. For each sample, analysis was performed using the average of positive/negative EMHC fibers from three pictures to be representative of the sample. **, *P* < 0.01; ***, *P* < 0.001.

To further investigate the role of PRMT1 in adult MSCs *in vivo*, we assessed the impact of its absence during regeneration. We injected cardiotoxin (Ctx) in tibialis anterior (TA) muscle of mice, causing necrosis of the fibers and triggering regeneration as previously described (19). After 21 days of regeneration, we harvested the injured and uninjured TA muscle from *PRMT1*^*FL/FL*^; − and *PRMT1*^*FL/FL*^; *Pax7*^*creERT2/+*^ mice. We first observed a gross size reduction of the TA from injured *PRMT1*^*FL/FL*^; *Pax7*^*creERT2/+*^ mice compared to uninjured and *PRMT1*^*FL/FL*^; − muscles (Fig. 6A). Further analysis revealed an ∼60% reduction of the cross section area in the *PRMT1*^*FL/FL*^; *Pax7*^*creERT2/+*^ mice 21 days after Ctx injection, suggesting that the muscle failed to regenerate after the necrosis induced by Ctx (Fig. 6A and B). Regeneration was impaired, as visualized by immunofluorescence, as smaller fibers were observed in *PRMT1*^*FL/FL*^; *Pax7*^*creERT2/+*^ mice, suggestive of a delay in the differentiation process (Fig. 6B). Interestingly, the loss of PRMT1 resulted in an increase in Pax7^+^ cells after Ctx injury (Fig. 6C), concurring with observations in MSC culture (Fig. 5C). Nevertheless, regeneration was impaired, supporting a requirement for PRMT1 in differentiation and maturation of the muscle progenitors. We then indexed the postinjury regeneration at 5, 10, and 15 days following Ctx injection. Cryosections were immunostained with embryonic myosin heavy chain, a marker of early regeneration (Fig. 6D and E). After 5 days, muscles from both wild-type and *PRMT1*^*FL/FL*^; *Pax7*^*creERT2/+*^ mice began the regeneration process, and we observed an equal percentage of embryogenic myosin heavy chain-positive (EMHC^+^) fibers from the two sets of mice (Fig. 6D and E). After 10 days, ∼50% of regenerating fibers were immature in *PRMT1*^*FL/FL*^; − mice versus ∼80% in *PRMT1*^*FL/FL*^; *Pax7*^*creERT2/+*^ mice. Fifteen days after Ctx injection, ∼80% regenerating fibers were EMHC^−^ in wild-type mice, whereas ∼80% of the fibers from PRMT1-deficient MSCs were EMHC^+^ (Fig. 6D and E). These results demonstrate the requirement of PRMT1 for proper myogenic differentiation and ultimately muscle regeneration.

## DISCUSSION

Muscle injury causes adult MSCs to exit quiescence and enter several rounds of self-renewal both to ensure the maintenance of the stem cell pool and to induce differentiation to ultimately repair damaged fibers (34). In the present study, we were interested in defining a role for PRMT1 in the modulation of MSC fate. We observed that PRMT1 did not affect the quiescence or activation of the MSCs but was required for the control of cell expansion and MSC fate decisions, since PRMT1-deficient MSCs repressed *MyoD* expression and had enhanced proliferation. Mice with PRMT1-null MSCs displayed an important skeletal muscle regeneration failure. Despite the regeneration impairment, we found that absence of PRMT1 in MSCs promoted their expansion during regeneration but repressed their differentiation. In searching for PRMT1 substrates, we identified the coactivator of Six1, Eya1, as a substrate. Eya1-deficient C2C12 cells had differentiation defects associated with *MyoD* repression. Taken together, these data suggest that methylation of Eya1 is required for proper muscle differentiation and muscle regeneration.

The decision for MSCs to self-renew, enter into proliferation, or return to quiescence is driven by complex gene networks and epigenetic events (35) that are still not fully understood. It is, however, well established that epigenetic programs govern a vast panel of biological processes, including tissue regeneration, stem cell differentiation, and aging (36). Since these events are heritable from the mother cells to the daughter cells and can be triggered by both internal and external cellular signals (37), it is fair to assume that they are essential for the stem cell fate decision and critical for the balance between MSC proliferation and differentiation. PRMT1 was reported as a critical regulator of hematopoietic stem cell fate decision and a key mediator of their self-renewal (38).

We report that Eya1 is a substrate of PRMT1. Eya1 is a phosphatase and a cofactor of the *Six* gene family (27, 29). Six proteins are required to drive myogenesis, as they regulate the expression of genes upstream of the myogenic regulatory factor genes, including *MyoD* (25, 26). Eya1/Eya2 double knockout mice are muscle-less, while Eya1 or Six1 mutants have strong myogenesis impairment (25). In adults, Eya1 has not been actively studied, but its requirement for *Six1* expression was shown to be responsible for muscle progenitor fate decision from slow- to fast-twitch fiber (27, 39). Importantly, it was shown that during organogenesis, Eya1 acts as a Six1 coactivator by interacting with Six1 and switching Six1 from repressor to activator (39). Following PRMT1 depletion, we observed a decrease in known Six1/Eya targets, *MyoD* and *Six1* mRNAs, as well as a decrease in the H3K4me3 activation mark, consistent with a defect in Eya1 recruitment at these promoter regions. Because Eya1 was shown to be required for Six1 presence (39), we hypothesize a model where PRMT1-mediated methylation promotes Eya1 recruitment at Six1 target genes. Six1 was also reported as a p53 repressor (40, 41), suggesting that Six1 could affect directly the cell cycle progression. The absence of Eya1 in C2C12 impaired differentiation and decreased *MyoD* expression, confirming that PRMT1 and Eya1 affect the same pathway.

The absence of MyoD expression in PRMT1-deficient MSCs explains their differentiation defects. MyoD is a master regulator and activator of the differentiation cascade, as it first induces expression of p21 to promote cell cycle arrest and then activates the expression of the differentiation termination factor myogenin (33). The absence of PRMT1 in MSCs promoted their expansion *in vivo* only during regeneration. A similar phenotype was observed by Tierney et al. (42) using Stat3-deficient MSCs, which resulted in an increase in their expansion but compromised their ability to differentiate, affecting their regeneration properties. It was also observed that transient inhibition of STAT3 enhances both the MSC expansion and muscle regeneration in aged muscles, suggesting that it counteracts the functional decline of the MSCs associated with age (42).

Recently, the role of arginine methylation in skeletal muscle regeneration has received lots of attention. PRMT5 was shown to be required for adult MSC proliferation but dispensable for embryonic myogenesis (23). PRMT7 depletion in MSC leads to premature senescence and is required to preserve their regenerative and self-renewing capacity (19). Asymmetrical dimethylation of Pax7 by CARM1 regulates self-renewal and preserves the pool of adult MSCs (22). Now we can add PRMT1 to the list of PRMTs involved in the control of the MSC proliferation and proper myogenic differentiation. The challenge will be to define the synergic roles that arginine methylation plays during MSC cell differentiation.

In conclusion, we report a role for PRMT1 in regulating several key transcription factor required for MSC fate, including Six1 and MyoD, through arginine methylation of Eya1. A better understanding of how to maintain MSC identity and how to control their expansion could help develop small molecules for *ex vivo* MSC expansion for therapeutic purpose.

## MATERIALS AND METHODS

### Animal generation and experiments.

All animal studies were performed according to approved protocols by the McGill University Animal Care Committee. The *PRMT1* conditional allele has been previously described by Yu et al. (6). These mice were backcrossed for >10 generations in the C57BL/6 background before interbreeding to obtain *PRMT1*^*FL/FL*^ homozygotes. For the generation of MSC-specific deletion, *PRMT1*^*FL/FL*^ mice were bred with the inducible mouse model, Pax7^CreERT2^ (44). Once the proper genotype was obtained, the mice were treated with a daily dose of 1 μg/25 g of 4-hydroxytamoxifen (OHT) for 5 days. Deletion of PRMT1 was verified by mRNA and protein analysis.

For regeneration experiments, TA muscles were injected with 10 μl of a solution of 10 μM Ctx. For injury studies, TA muscles were harvested and frozen in isopentane cooled in liquid nitrogen and stored at −80°C prior to sectioning. At the same time, abdominal muscles and diaphragm were extracted for MSCs using magnetically activated cell sorting (MACS)-based isolation.

### Tissue culture and transfection.

Myoblast progenitor C2C12 cells were cultured in Dulbecco modified Eagle medium (DMEM) containing 20% (vol/vol) fetal bovine serum (FBS) and 1% (vol/vol) penicillin-streptomycin. Once C2C12 cells reached confluence, differentiation was induced in DMEM supplemented with 2% FBS. HEK293T cells were cultured in DMEM containing 10% FBS and penicillin-streptomycin.

Cells were transfected with small interfering RNAs (siRNAs; Dharmacon, Lafayette, CO) using Lipofectamine RNAi MAX (Invitrogen) according to the manufacturer's instructions. Plasmid DNA was transfected using Lipofectamine 2000 (Invitrogen).

Satellite cells were isolated from freshly extracted skeletal muscles using the satellite cell isolation kit (Miltenyl) according to the manufacturer's protocols.

Isolated satellite cells were cultured in serum-rich medium (39% DMEM, 39% DMEM–F-12, 20% FBS, and 2% UtroserG [Pall Biosciences]) for proliferation experiments and in serum-low medium for differentiation experiments (DMEM–F-12 supplemented with 5% horse serum) at 37°C in a 5% CO_2_ humid atmosphere.

### Tissue processing, immunostaining, and microscopy.

TA cryosections were fixed with formalin 10% (Sigma) at room temperature for 15 min and permeabilized in cold methanol at −20°C for 6 min, except for EMHC staining, for which sections were not fixed.

Immunostainings of TA sections and muscle stem cells were performed with primary antibodies anti-PRMT1 (made in-house), anti-Pax7 (DSHB), anti-Myf5 (Santa Cruz), anti-troponin T/C (Santa Cruz), anti-EMHC (DSHB), antilaminin (Sigma), or anti-Ki67 (AbCam) overnight (O/N) at 4°C and with secondary antibodies Alexa Fluor 488 and 546 1 h at room temperature. Samples were all mounted in Immu-Mount (Thermo Scientific) containing 4′,6-diamidino-2-phenylindole (DAPI; VectaShield). Images were acquired with a Zeiss Axio Imager 2 microscope and analyzed with Axio imager software, using the same settings for all image sets. Analyses of satellite cells and TA sections were performed in a double-blind manner. Image channels were decomposed using ImageJ and postprocessed in Photoshop for proper curve levels to increase image quality without altering the content of the original image. TA were imaged using a Nikon7100D and postprocessed in Photoshop for proper white balance.

### Immunoblotting and immunoprecipitation.

Cell lysates (50 mM HEPES [pH 7.4], 150 mM NaCl, 1% Triton X-100) and freshly added EDTA-free protease inhibitor cocktail (Roche) were immunoblotted with antibodies against PRMT1 (made in-house), MyoD (Santa Cruz), ASYM26 (Millipore), FLAG-M2 (Sigma), and Myc (Sigma). Protein extracts were resolved by SDS-PAGE, transferred to a nitrocellulose membrane using an immunoblot TurboTransfer system (Bio-Rad), blocked for 1 h at room temperature in Tris-buffered saline with Tween 20 (TBST)–5% milk and incubated with primary antibody, followed by incubation of secondary antibodies conjugated to horseradish peroxidase (Sigma-Aldrich). Western Lightning Plus ECL from PerkinElmer was used for chemiluminescence detection.

For immunoprecipitations, 48 to 72 h after transfection, cells were lysed in lysis buffer (1% Triton X-100, 150 mM NaCl, 20 mM Tris-HCl [pH 8.0]) freshly complemented with EDTA-free protease inhibitor cocktail (Roche). Supernatants were collected and incubated with primary antibodies for 1 h on a spinning wheel, and then 25 μl of FLAG-M2 (Sigma) slurry was added and incubated at 4°C for 30 min on a spinning wheel. The beads were then washed 5 times with lysis buffer and boiled with 4× Laemmli buffer prior to immunoblotting.

### Chromatin immunoprecipitation.

Chromatin immunoprecipitation was performed as described previously (17). The antibodies used were Eya1 (AbCam), Six1 (Sigma), H3K4me3 (AbCam), and H3 (AbCam).

### RNA extraction and RT-qPCR.

RNA was extracted using TRIzol reagent (Life Technology) in accordance with the manufacturer's protocol. RNA was normalized and used for RT-qPCR using EvaGreen (Bio-Rad) and the 7500 Fast real-time PCR system (Applied Biosystems). Transcript levels were normalized to an average of *GAPDH*, *TATA Binding Protein* (*TBP*), and RNAr18S and then to the control condition. RT-qPCR assays were performed using the MIQE guidelines.

### Peptide arrays.

Peptide arrays designed *in silico* based on predictions and the substrate preference of PRMTs were synthesized as described before (43). Peptides from histones H2B and H4 were used as controls. The membranes harboring the quilted peptides were incubated with 1 μg recombinant glutatione *S*-transferase (GST)–PRMT1, [^3^H]*S*-adenosylmethionine in 1× PBS). The filter was washed and visualized by fluorography.

### Statistical tests.

Statistical significance was assessed using Prism5 software via Student's *t* test (unpaired, 95% confidence interval [CI]) or the analysis of variance (ANOVA) test (one-way or two-way ANOVA), followed by Tukey's *post hoc* test (95% CI). Error bars show standard deviations (SD). Values with *P* values of <0.05 were considered statistically significant.
